# MILANO – custom annotation of microarray results using automatic literature searches

**DOI:** 10.1186/1471-2105-6-12

**Published:** 2005-01-20

**Authors:** Ran Rubinstein, Itamar Simon

**Affiliations:** 1 Department of Molecular Biology, Hebrew University – Hadassah Medical School, Jerusalem 91120, Israel

## Abstract

**Background:**

High-throughput genomic research tools are becoming standard in the biologist's toolbox. After processing the genomic data with one of the many available statistical algorithms to identify statistically significant genes, these genes need to be further analyzed for biological significance in light of all the existing knowledge. Literature mining – the process of representing literature data in a fashion that is easy to relate to genomic data – is one solution to this problem.

**Results:**

We present a web-based tool, MILANO (Microarray Literature-based Annotation), that allows annotation of lists of genes derived from microarray results by user defined terms. Our annotation strategy is based on counting the number of literature co-occurrences of each gene on the list with a user defined term. This strategy allows the customization of the annotation procedure and thus overcomes one of the major limitations of the functional annotations usually provided with microarray results. MILANO expands the gene names to include all their informative synonyms while filtering out gene symbols that are likely to be less informative as literature searching terms. MILANO supports searching two literature databases: GeneRIF and Medline (through PubMed), allowing retrieval of both quick and comprehensive results. We demonstrate MILANO's ability to improve microarray analysis by analyzing a list of 150 genes that were affected by p53 overproduction. This analysis reveals that MILANO enables immediate identification of known p53 target genes on this list and assists in sorting the list into genes known to be involved in p53 related pathways, apoptosis and cell cycle arrest.

**Conclusions:**

MILANO provides a useful tool for the automatic custom annotation of microarray results which is based on all the available literature. MILANO has two major advances over similar tools: the ability to expand gene names to include all their informative synonyms while removing synonyms that are not informative and access to the GeneRIF database which provides short summaries of curated articles relevant to known genes. MILANO is available at .

## Background

In the post-genomic era, biologists encounter a flood of information derived mainly from microarray experiments. The blessing of this wealth of information is accompanied by a great difficulty in identifying the biologically significant findings, which are often embedded in irrelevant information. Currently, there are several approaches to deal with this problem. One approach is to identify a category of genes which is overrepresented in the microarray output. This approach can be carried out using the Gene Ontology project (GO) which describes gene products in terms of their associated biological processes, cellular components and molecular functions [1]. The advantage of this approach is that it can be easily automated and thus can be used for quick screening of large outputs. On the other hand, this approach limits the analysis to the structure of the GO project and thus does not support the desire of many researchers to customize their analysis. A second approach involves searching the literature for information about each of the genes on the list. Although this approach is comprehensive, it suffers from many downsides: it is time consuming; there is no systematic way to integrate the information learned about each gene; usually one gets distracted with seemingly interesting comparisons early on during the literature search and thus does not give the genes at the end of the list the same weight that was given to genes that appear at the top of the list; there are multiple names and symbols for each gene and thus it is hard to extract the literature information for any particular gene since each author may refer to it differently. A third approach entails curated databases that have gathered all the known information pertaining to each gene. This approach is limited by the quality of the curation process. For example for studying the yeast *Saccharomyces cerevisiae*, there are excellent curated databases, such as the Yeast Proteome Database [2] and the Saccharomyces Genome Database [3], which contain all the known information about each gene. On the other hand in other organisms the curation procedure is at a less advanced stage and thus the information contained in the curated databases is still partial.

We have developed an analysis tool that combines the advantages of all the mentioned approaches and overcomes some of the disadvantages. Our tool (MILANO – Microarray Literature-based Annotation) uses an automatic search of literature databases for performing custom annotation of the list of genes obtained from a microarray output. This is done by generating dynamic annotations for genes, built according to terms provided by the researcher. The program receives as input a list of gene identifiers obtained from any microarray experiment and a set of custom search terms. The program expands each gene identifier to its informative synonyms and searches literature databases for co- occurrences of every gene on the list with each of the custom terms. The program's output is an annotation table with the numbers of publications for each gene-term combination (hit-counts). This novel annotation format can be easily used within a web browser or a spreadsheet program to quickly identify genes within the list that are related to the terms provided by the researcher, and may be easily extended, as every hit-count in the annotation is a hyperlink to the query's results. The great advantage achieved by this method over standard static annotations, such as Gene Ontology (GO) annotations, is that the annotations are generated based on terms provided by the researcher, and therefore help in addressing the specific scientific question the researcher is pursuing.

The program is able to search two literature databases, GeneRIF [4] and Medline [5]. GeneRIF contains ~90,000 short summaries of curated articles relevant to known genes. An initial search of the microarray results against the GeneRIF database provides results within minutes and is easily evaluated, thereby providing immediate insights to the microarray results. This search is followed by a comprehensive Medline search via Pubmed, allowing the identification of more subtle biological insights.

To demonstrate the power of this strategy, we have analyzed a list of 148 genes affected by over-expression of p53 [6]. Our analysis assisted in retrieving from the list 11 known p53 targets, which are all the known targets in the list, and in identifying within the p53-affected genes a subset of putative p53 target genes that are known to be involved in apoptosis (43 genes), in cell cycle arrest (21 genes), and in Cancer (48 genes) as shown in Figure 3. This example demonstrates the usefulness of our tool in narrowing down microarray results to a small list of genes involved in a specific biological activity.

## Implementation

### Web Interface

**Figure 1:**
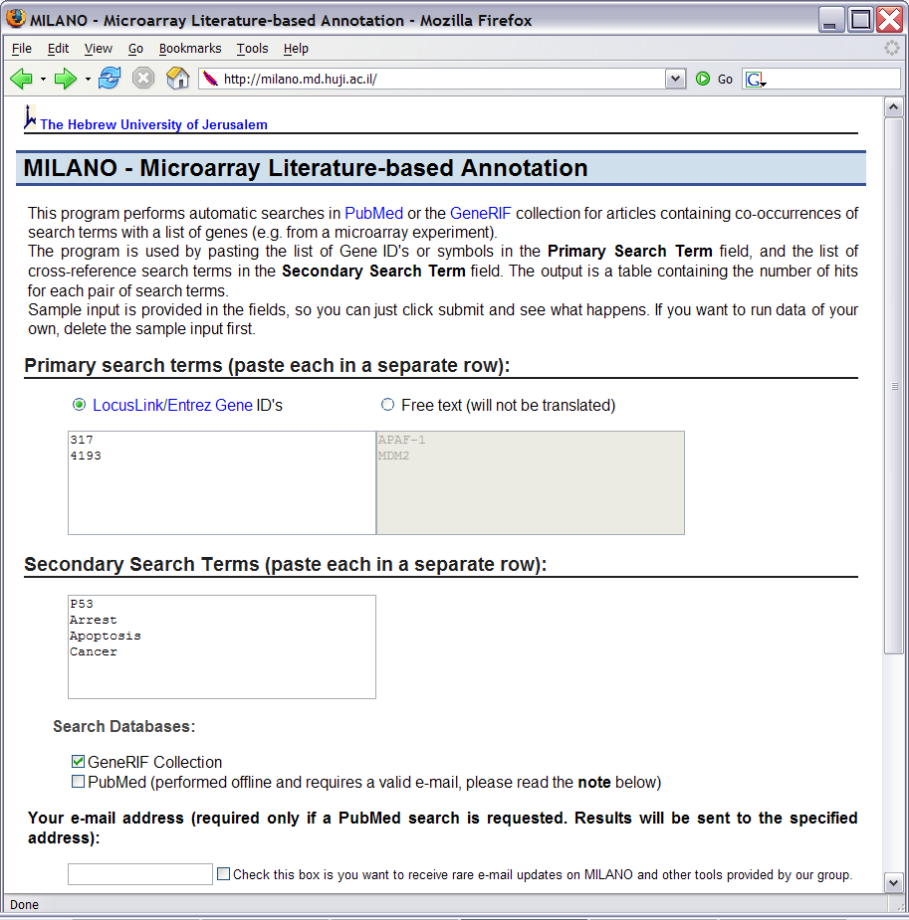
The MILANO data input page .

MILANO is accessed through a familiar web form (Fig 1A). A CGI (Common Gateway Interface)-based Perl [7] program is executed on submission, which creates the combined Boolean searches for the requested databases. The user can decide whether to provide gene symbols directly, or provide LocusLink/Gene numbers, which are expanded to synonyms as described below. Results, formatted as an HTML table, are displayed immediately on-screen for GeneRIF searches and sent by e-mail for Pubmed searches.

### Synonym expansion

Gene aliases are collected from the LocusLink database file, downloaded from the NCBI ftp server [8]. We use an awk [9] program to extract gene symbols, aliases and product names. The alias collection is then processed by a Perl program that removes symbols that are shorter then three characters or that appear in a 23,000-word English dictionary, enhanced for scientific terms. This database is stored in a fashion than enables us to extract processed aliases for a gene by its LocusLink number.

### Pubmed searches

Pubmed searches are performed by a Perl program which uses the NCBI eutilities esearch web service for accessing the Pubmed database [10]. There are limitations on when and how often we can query the NCBI server, so we integrated into the program a mechanism that makes sure that is does not make more than one query every three seconds. The Generic NQS (Network Queuing System) [11] ensures that jobs that include more than 100 queries run only between 9 p.m. and 5 a.m. ET.

### GeneRIF searches

The GeneRIF collection is automatically downloaded weekly from the NCBI ftp server [12], and processed by a Perl program to include gene symbols from the synonym expansion database into every GeneRIF. The database is then indexed by a database server (SRS 7.1.3, Lion Bioscience AG), which provides a query interface for counting and displaying GeneRIF entries.

## Results

### Expanding the search terms

One of the major problems in the automation of literature searches is the ambiguity in gene names [13]. Multiple names are used in the literature for any specific gene and thus it is not straightforward to define the Medline query that will find most of the relevant information on a gene. In order to overcome this problem we used the LocusLink database [14] to expand any gene symbol to all its synonyms. We also included in the expanded form the gene product name since many genes are mentioned in the literature by their product name and not by one of their symbols (for example most of the citations for the beta actin gene can be found by searching the Medline with the term "beta actin" and not with its official symbol "*ACTB*"). Although expansion of the search terms is a useful tool to increase the number of articles retrieved for each gene it also adds many irrelevant articles due to the fact that some of the gene symbols are not informative as Medline query terms. For example one of the aliases of the gene aquaporin_1 is *CO*, a term that is mostly mentioned as an abbreviation for Carbon mono-oxide, and one of the aliases of the gene CD36_antigen is *FAT*, which is found in over 100,000 articles, unrelated to CD36. In order to diminish this problem we filtered out from the list of gene symbols any term that was shorter than three characters and any term that is an English word. In order to check our name expansion strategy we conducted a Medline search for 16862 well-known human genes (all the genes that have an NM number indicating the identification of their full length mRNA), using three search strategies: using only the official symbol for each gene (Symbol), using the official symbol together with all its aliases and the gene product (Expanded) and using only the informative terms (Filtered). Using the Expanded search allowed the identification of literature information on about ~1900 additional genes over a query using the official symbol only (Table 1). Using the Filtered search terms allowed this addition without adding significantly to the number of queries that returned non-reasonable results. In addition to expanding the number of genes that were found in the literature, the Filtered search terms also increased the number of articles found per gene (from an average of 198 articles per gene found by searching with the symbol alone to an average of 451 articles per gene when searching with the filtered terms). These results indicate that our gene name expansion strategy achieves a higher percentage of relevant literature for each gene while limiting the addition of irrelevant information.

### Conducting automatic literature searches

**Figure 2:**
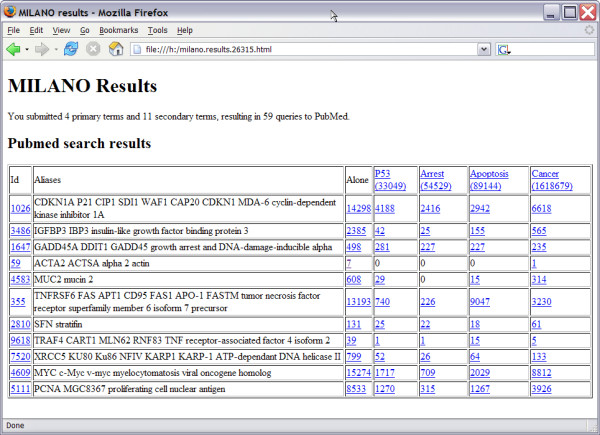
An example of a result of MILANO search using a short list of gene symbols that were expanded by the program to include all their informative synonyms versus p53 related terms. All reported numbers are hyperlinked and will initiate a new search for that specific term combination.

After expanding the search terms, MILANO performs an automatic search of literature databases, and retrieves the number of hits each query returned. MILANO performs Boolean searches in which one can search for co-occurrence of each of the primary terms (the expanded gene name) with user defined secondary terms (Figure 1). The program's output is a table (Figure 2) containing the number of publications for each gene-term combination (hit-counts). This table could serve as an annotation table, because the number of publications reflects the relationship between the genes to the secondary term used. For example a gene that has a role in DNA damage will appear in more articles about "DNA damage" or "gamma irradiation" than unrelated genes.

In order to assist in further evaluation of the results, we have built the annotation table such that each number in the table is a hyper-link to the literature database and thus clicking on it will perform this specific search again and will open a window containing the actual abstracts found by this combination of search terms.

### Literature databases supported by the program

The MILANO program can search two databases (Figure 1) – the full Medline database, currently containing more than 12,000,000 references, and the GeneRIF database that contains more than 90,000 short summaries of curated articles relevant to known genes. There are several advantages in using the GeneRIF database over the full Medline: the searches are quick and the results are obtained within minutes; each article is summarized by a sentence or two, reducing the amount of information that needs to be read; the curation procedure extracts from the papers only the information relevant to the gene, minimizing the cases in which two terms appear in the same abstract but are not related to each other; the GeneRIF entries are based on the full text of the articles and not only on the abstracts. However, since the curation procedure is an on-going process, the coverage of this database is only partial and thus information is missing and can be found only by performing a Medline search. For that reason our tool allows a combined search strategy in which both databases can be searched simultaneously. The GeneRIF database provides results within minutes and is easily evaluated, thereby providing immediate insights to the microarray results. In parallel a comprehensive Medline search can be done. Although this search takes longer and its results obtained by email, it allows the identification of more subtle biological insights.

### P53

To demonstrate the power of our literature-based annotation strategy, we analyzed a list of 148 genes affected by over-expression of p53 [6]. This list of genes was obtained by microarray experiments and nicely demonstrates the difficulty of microarray analysis since it contains many putative p53 target genes and their relevance to p53 cellular activity is not clear.

**Figure 3:**
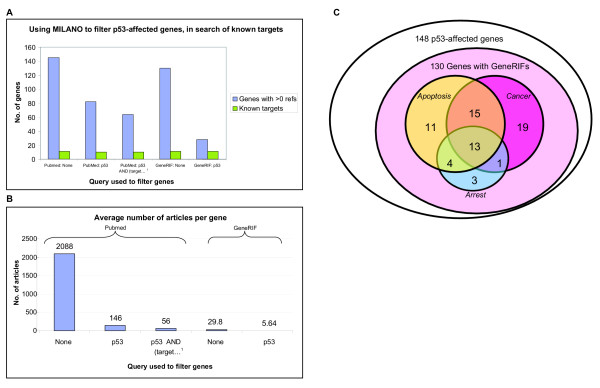
**Analysis of a list of genes affected by p53 overproduction. **A. The number of genes remaining after filtering the p53-affected genes with terms intended to reveal known p53 targets. B. Average number of articles per gene in the different queries. C. Venn diagram depicting the different functions of p53 affected genes as reflected by a GeneRIF search. ^1^Search term is "p53 AND (target OR transcriptional OR activation OR repression)"

Our first aim was to identify the known p53 target genes that were affected by p53 overproduction in this experiment. By using specific secondary terms, we were able to trim down the list of 148 genes to a much shorter list of genes highly enriched for known p53 target genes (Figure 3A). In order to evaluate the number of target genes that were missed by our annotation strategy, we manually compiled a list of all known p53 target genes, ~60 genes. Eleven of these 60 genes were represented in the list of genes affected by over-expression. Our automatic annotation strategy found all of them. Moreover, the use of MILANO reduced the amount of articles per gene from an average of 2088 articles per gene in the initial list to 56 articles per gene in the limited list (Figure 3B). The p53 example also demonstrates the usefulness of searching the GeneRIF curated database in which the use of the secondary term p53 allows filtering out most of the irrelevant genes without losing any known target gene (Figure 3A).

P53 is involved in apoptosis, cell cycle arrest and cancer. It is interesting to find out which of the genes affected by p53 is involved in these processes. Using MILANO we easily identified genes known to be involved in these processes (Figure 3C), which helped the process of analyzing the microarray data.

### Comparison with other tools

Recently, few literature mining tools has been developed, using a similar approach to the one presented here [15-17], however all of them suffer from the problem of inappropriate use of primary search terms. In order to demonstrate the advantage of using MILANO over the other tools, we have performed a comparative analysis of all these tools by looking at their performance on the 11 known p53 target genes described above. The software were run with these 11 genes as the primary search terms and "P53" as the secondary term and reported the number of occurrences of those terms. The results that are summarized in table 2 demonstrate that MILANO-GeneRIF search was the only method that revealed connections between all 11 genes and p53 and that the MILANO-Medline search gave the most comprehensive search results. PubMatrix [15] does not expand the primary search terms and thus it misses many literature occurrences. This problem is best demonstrated by its poor performance on the CDKN1A gene which is one of the most studied targets of p53. The synonym expansion methods used by MicroGENIE [16] improved the results regarding the CDKN1A gene, but missed the SFN gene completely, and gave non-informative synonyms to XRCC5 and TRAF4 ("Ku" and "TNF" respectively). BEAR GeneInfo [17] did not perform synonym expansion correctly for CDKN1A, and gave non-informative synonyms for PCNA and TRAF4 ("cyclin" and "h. mln62 mrna" respectively). When we attempted to analyze the full data set of 148 genes, some of the compared tools failed to give results due to errors.

## Discussion

MILANO is a simple and intuitive literature search tool. It allows automatic Medline and GeneRIF searches followed by a quick survey of the results. Using this tool dramatically reduces the time needed to query literature databases. Moreover, due to its systematic nature, it assists in treating the 1^st ^and the 100^th ^query in an unbiased manner. The MILANO program uses all the published information for the annotation of each gene according to its co-occurrence in the literature with a user defined secondary search term. These features of MILANO makes it especially suitable for analyzing microarray results, since it can be used to annotate the results with terms defined by the user and not limited by preset terms such as the GO terms based annotation.

We have demonstrated the power of our program by the analysis of a list of 148 genes that were deregulated in cells that overproduced the p53 tumor suppressor gene [6]. Frequently one of the first tasks in microarray data analysis is to determine the overlap between new results and results expected based on the literature. For example in analyzing the list of genes induced by over expression of p53 one expects to find known p53 target genes. Thus, we applied our automatic literature search tool in order to answer this question. We found that use of this tool dramatically shortens the time needed for such an analysis by allowing the researcher to focus on a relatively small subset of potential target genes and by reducing the amount of literature relevant to each gene (Figure 3). Our tool was also found useful in automatically sorting the target genes into functional groups. Based on the knowledge of p53 cellular functions we defined secondary search terms that fit p53's main activities – apoptosis and cell cycle arrest [18]. Using these terms allowed the quick identification, from the primary list, of a subset of genes that were not known to be involved in those processes and thus may be interesting for further research (Figure 3C).

Several literature mining approaches have been developed to integrate multiplex biological datasets into the context of published medical literature. A good example of such an approach is the PubGene program [19], which searches for literature co-occurrences of gene names in order to build a network among the genes. PubGene is useful for quickly realizing and viewing known relationships between genes, but it does not assist in annotating gene lists. To this end one needs an automatic literature searching tool that allows the use of flexible secondary terms with which co-occurrences are counted. Recently such tools have been built. PubMatrix [15] allows automatic Boolean searches to be performed on Pubmed using any list of primary and secondary terms. This tool carries out the search on the exact terms entered by the user thus in order to apply it to the analysis of microarray data, one has to translate each of the enriched spots to a name suitable for a Medline search. Two other tools – microGENIE [16] and BEAR GeneInfo [17] uses a very similar approach but in order to make it more compatible to microarray analysis, they allow the use of gene identifiers as input and provides the needed translation to gene names. During the translation the gene name is expanded to include its synonyms. All of these tools have improved the ability of researchers to quickly use the published literature to annotate lists of genes. However, they suffer from the limitations of any literature data search tool; the ambiguity of gene names and the partial information that can be retrieved by limiting the literature searches to abstracts [13].

MILANO's aim is to further improve the literature based automatic annotation approach by adding two essential features that address these limitations:

### Smart synonyms

Each gene symbol is expanded to all its aliases, while removing non-informative terms, and the gene product name is added to the query. This addresses the synonym problem, while omitting many of the irrelevant results, thus reducing the polysemy problem (words with multiple meanings). The advantage of our synonym expansion scheme over the existing tools is demonstrated by the comparison presented in table 2.

### The GeneRIF database

In contrast to the existing tools, MILANO is able to search not only the Medline database, but also the GeneRIF database, which contains short summaries of articles relevant to known genes. The curation of GeneRIF is done by the National Library of Medicine's MeSH indexing staff, who have advanced degrees in the life sciences and use the full text of articles for the indexing process [4]. Using this database reduces the limitations of relying only on abstracts and aids in finding only relevant information about each gene. Nevertheless, the GeneRIF database suffers from the problems of all manually curated databases; it is partial and contains mistakes and biases introduced by the curation team. However, our ability to identify all of the p53 target genes within a group of p53-affected genes by using the GeneRIF database alone (Figure 3) demonstrates that, at least for well annotated genes, using such a database may be the ideal solution for annotating microarrays results. The quality of GeneRIF-based annotation depends on the amount of information entered for each gene in the GeneRIF database, which for many genes is insufficient (data not shown). However, its performance will improve as more information is incorporated into this database and we believe that in the future it will become the preferred annotation tool. Meanwhile, we recommend using MILANO for performing combined searches; searching the GeneRIF database provides quick results and searching the full Medline database allows a broader view that is not limited by the curation procedure.

## Conclusions

We present MILANO , a literature mining tool that can help in annotating microarray results in light of all available literature using experiment-specific terms. In designing MILANO we focused on the accuracy of the search results by providing two novel features: i) Expansion of gene names to include in the literature searches all their informative synonyms, while removing non-informative synonyms; ii) Searching two literature databases – Medline and GeneRIF. While Medline encompasses all the literature and provides the most comprehensive results, it also contains many irrelevant articles. GeneRIF provides a subset of Medline articles that are relevant to known genes and thus avoids most of the irrelevant results often found in Medline searches.

The usefulness of MILANO is demonstrated by the automatic analysis of a list of 148 p53 target genes. The use of literature mining dramatically reduced the time and effort required for a task such as identifying the known p53 target genes within this list. A search in GeneRIF immediately discovered the full list of target genes, with no false hits.

## Availability

All software and databases are freely available and may be executed online at our web site: . The author will provide data, scripts and programs used on demand. We encourage users to install the software on their own servers, as we provide no assurance to the privacy or accuracy of the results.

## Authors' contributions

RR designed and programmed the software. IS managed the project and drafted the manuscript.

## Figures and Tables

**Table 1:** Summary of Medline hit counts for all the full length mRNA genes (16,862 genes) using different search strategies.

Type of primary term^a^	Positive results^b^	Non reasonable results^c^	Articles per gene^d^
Symbol	10,045	20	198
Expanded	12,028	140	817
Filtered	11,910	22	451

^a^The Medline search was conducted using three searching strategies: Symbol refers to a search in which each gene was represented by its official symbol; Expanded refers to searches in which each gene was represented by the gene symbol, all its synonyms and the official gene product name; Filtered refers to searches in which non informative names were filtered out of the expanded list.

^b^Number of queries that returned at least one result.

^c^Number of queries that returned more than 33,000 results. We used 33,000 as a rough estimate of non reasonable results based on the fact that some of the most investigated genes, like p53, appear in less than 33,000 abstracts.

^d^The average number of abstracts per gene counting only genes that appeared at least once and did not appear in more than 33,000 abstracts.

**Table 2:** Comparative analysis of literature mining tools. Eleven known p53 target genes were analyzed using five methods. The numbers represent the number of reoccurrences of each gene with the term "P53".

**Gene Id**	**Primary Symbol**	**MILANO – GeneRIF**^a^	**MILANO – Medline^a^**	**PubMatrix^b^**	**BEAR GeneInfo^a^[17]**	**MicroGenie^c^**
1026	CDKN1A	74	4180	49	50	3058
1647	GADD45A	7	281	21	45	313
2810	SFN	2	25	25	25	0
3486	IGFBP3	1	42	36	42	36
355	TNFRSF6	25	740	418	43	707
4583	MUC2	2	29	29	29	29
4609	MYC	21	1715	1715	1715	1715
5111	PCNA	13	1269	1173	3671	1173
59	ACTA2	1	0	0	0	0
7520	XRCC5	1	52	42	42	583
9618	TRAF4	2	1	1	1570	326

^a^The search was performed with LocusLink ids as the primary search terms.

^b^The search was performed with the primary gene symbols as the primary search terms.

^c^The search was performed with UniGene ids as the primary search terms.
